# Anxiety symptoms following conception through assisted reproductive technology: a systematic review

**DOI:** 10.3389/fpsyt.2026.1709200

**Published:** 2026-07-20

**Authors:** Nicole L. Davies, Lauren Carson, Roxanne C. Keynejad

**Affiliations:** 1 Department of Psychology & Neuroscience, Temple University, Philadelphia, PA, United States; 2 Department of Psychological Medicine, Institute of Psychiatry, Psychology & Neuroscience, King’s College London, London, United Kingdom; 3 UK Biobank Ltd, Stockport, United Kingdom; 4 King’s Women’s Mental Health, Health Service and Population Research Department, Institute of Psychiatry, Psychology & Neuroscience, King’s College London, London, United Kingdom

**Keywords:** anxiety, assisted reproductive technology, fertility treatment, *in vitro* fertilization, perinatal mental health

## Abstract

**Background:**

The use of assisted reproductive technology (ART) to aid conception is increasing worldwide, yet little research has explored psychological outcomes following ART conception.

**Objective:**

To systematically review the prevalence and mean anxiety symptom scores among women who conceived following ART, from confirmed pregnancy until 12 months postpartum, compared to women who conceived without ART (spontaneous conception, SC).

**Methods:**

We searched MEDLINE, PsycINFO, and EMBASE from their start dates to 3^rd^ June 2024 using a piloted search combining keywords related to ART, anxiety symptoms, pregnancy or the postnatal period, using Boolean operators. We extracted data on prevalence and mean anxiety symptom scores in the ART and SC groups, along with study characteristics.

**Results:**

Twenty studies met inclusion criteria, comprising cohort (n=15), case-control (n=1), and cross-sectional (n=4) designs. Most studies measuring symptoms during the first (n=3) and second trimester (n=8) reported non-significant differences, apart from one second trimester study which reported elevated pregnancy-specific anxiety symptoms in the ART group. Studies measuring symptoms during the third trimester (T3) (n=16) and postnatal period (n=8) reported mixed findings of higher (T3:n=4; postnatal:n=2), lower (T3:n=1; postnatal:n=2), and no significant differences (T3:n=9; postnatal:n=4) anxiety in the ART group relative to SC. Two third trimester studies reported higher pregnancy-specific anxiety but lower or equivalent generalized anxiety symptoms in the ART group.

**Conclusions:**

While some studies reported elevated anxiety symptoms following ART, particularly in late pregnancy and postpartum, findings remain mixed, with others indicating no differences or lower symptom levels. Further longitudinal research is warranted to better understand the postpartum experiences of women with fertility difficulties.

## 1 Introduction

The use of assisted reproductive technology (ART) is increasing worldwide, with up to 7.9% of births in European countries resulting from ART in 2018 (1). ART refers to medical procedures designed to treat infertility and other difficulties with spontaneous conception (SC) (2). *In vitro* fertilization (IVF) accounts for approximately 99% of ART procedures in the United States (3) and has led to over eight million live births worldwide (4). The increasing use of ART globally is in part attributable to the postponement of childbearing to later years in many high-income countries (5). ART also offers lesbian, gay, bisexual, transgender, and queer (LGBTQ+) individuals and couples the opportunity to become genetic parents (6). Given its rapid expansion, closer attention is warranted surrounding psychological sequelae associated with ART exposure.

Although beneficial in assisting with conception, ART is expensive and does not always lead to pregnancy and subsequent live birth (7). For example, each cycle of IVF in the United States (US) averages between $12,000 and $17,000 (8). In addition to financial costs, ART is associated with physical and psychological risks. ART often entails painful, time-consuming daily injections to stimulate ovulation, regular trans-vaginal ultrasound scans, and procedures including oocyte retrieval under sedation (9).

Preparation for IVF is associated with a high incidence of anxiety disorders (10). Heightened anxiety disorder rates can persist throughout the treatment process (11). Further, increased anxiety symptoms have been reported during specific treatment stages. For example, in a cohort of 247 couples undergoing IVF embryo transfer in China, 44.9 % scored  ≥ 50 on the Self−Rating Anxiety Scale on the day of the serum human chorionic gonadotropin pregnancy test, compared to 29.9 % at cycle entry and 17.8 % four days post−transfer (12). Current literature reports mixed findings surrounding whether anxiety during ART impacts conception rates (13, 14). Additionally, a large Australian cohort study highlighted that women with fertility problems were more likely to experience anxiety and depressive symptoms after childbirth (15). Further, in an assessment of 159 women who conceived using ART at a fertility center in Taiwan, average pregnancy−specific stress scores (assessed via the Pregnancy Stress Rating Scale) increased from 83 to 88 from conception to full term (16).

After assisted conception, babies are at an increased risk of congenital anomalies, being born small for gestational age, and having abnormal hormonal and cardio-metabolic profiles persisting into adolescence (4, 17, 18). ART also increases the risk of multiple pregnancy, which is associated with complications such as fetal growth restriction, preterm birth, pregnancy-induced hypertensive disorders, and gestational diabetes (19).

Maternal distress during pregnancy is associated with adverse obstetric and neonatal outcomes. A review by Grigoriadis and colleagues (20) found that antenatal anxiety, measured as a diagnosed anxiety disorder or clinically elevated anxiety symptoms on validated scales, was associated with preeclampsia, sleep disorders, low birth weight babies, and neonatal abnormalities. Notably, an emerging body of research into the developmental origins of health and disease stemming from the Barker Hypothesis (D. 21) highlights how prenatal maternal stress (PNMS) can alter the fetal environment and thus subsequently contribute to a cascade of lasting physical and psychological risk trajectories (22, 23). PNMS has been connected to long-term outcomes such as childhood asthma and later schizophrenia spectrum disorders (23–25). Further, prenatal maternal anxiety is associated with lasting internalizing difficulties in offspring (E. D. 26, 27). For example, in the Avon Longitudinal Study of Parents and Children (ALSPAC) cohort, each one−point increase in second−trimester maternal anxiety symptoms was associated with a 7 % increase in the odds of an anxiety−disorder diagnosis at age 18 years, and no comparable paternal associations were found (28). While causal inference is limited by reliance on animal models and observational designs, identifying perinatal stressors is important for developing services to optimize maternal and neonatal health during the perinatal period.

Recent literature on mental health outcomes after ART has typically prioritized the examination of perinatal depression over anxiety symptomatology (29). A recent meta-analysis reviewed postpartum depressive symptoms (PDS) within one year of delivery in an ART sample, cautiously noting a surprisingly lower incidence of PDS compared to women with SC, and emphasizing the need for better-designed trials to reveal a potential association (29). Notably, this was the first meta-analysis to examine the relationship between ART and PDS and did not address anxiety. A series of prior reviews has focused on distress during the ART process and the association with treatment outcomes (30, 31) and affective symptoms following unsuccessful ART (32), leaving anxiety symptoms after successful conception largely unexplored. One prior systematic review addressed psychological stress and adjustment in ART-conceived pregnancies and found greater pregnancy-specific anxiety but inconsistent evidence of generalized anxiety symptoms (33). Despite its novel contribution to the post-ART evidence base, the review lacked an SC control group, did not synthesize data on symptoms by gestational stage, omitted the postpartum period entirely, and covered studies only published between 2000 and 2014 (33).

Thus, to the authors’ knowledge, no systematic review to date has compared anxiety symptom trajectories across pregnancy and the first postnatal year in post-ART versus SC samples. The objective of this systematic review was to synthesize the available evidence regarding the relationship between ART conception and perinatal anxiety symptoms relative to SC. The primary outcome was the prevalence of anxiety symptoms or mean anxiety symptom scores among pregnant and postnatal women^1^ who conceived with ART, relative to SC.

## 2 Methods

### 2.1 Literature search strategy

The search strategy followed Preferred Reporting Items for Systematic Review and Meta-Analysis Protocols (PRISMA-P) guidelines for systematic reviews. See the PRISMA-P checklist in the appendix for further details. The first author (ND) conducted a systematic search in June 2024 of MEDLINE, EMBASE, and PsycInfo databases through the King’s College London OVID platform. All databases were searched from their start dates to 3 June 2024. The only limit applied to each search was that studies needed to enroll human participants. Only published, peer-reviewed articles available in English were considered for this review, as translation resources were not available.

### 2.2 Eligibility criteria

Eligible studies were required to be peer-reviewed cross-sectional, cohort, or case-control studies published in English, which reported either (1) the prevalence of anxiety symptoms or (2) a mean score on a validated anxiety symptom scale in at least some pregnant or postnatal participants who had received ART prior to the current conception. This review was guided by the Population, Intervention, Comparison, and Outcome (PICO) framework, as outlined below.

#### 2.2.1 Population

Eligible studies were required to enroll participants who were pregnant and/or up to 12 months postpartum.

#### 2.2.2 Exposure

The exposure of interest was conception using any form of ART.

#### 2.2.3 Comparison

Each study needed to report prevalence or mean anxiety symptom scores, with results disaggregated by ART versus SC.

#### 2.2.4 Outcome

Anxiety symptoms (either self-reported or observer-rated) had to be measured on a validated scale at any point during pregnancy and/or up to 12 months after birth.

### 2.3 Search terms

Key words were combined with MESH terms for concepts of “anxiety” AND “perinatal” AND “ART” using Boolean operators. For further details, see the list of search terms in the appendix.

### 2.4 Study selection

The screening process was conducted in the Covidence systematic review management software (34). All records identified in MEDLINE, PsycInfo, and EMBASE were imported into Covidence, where duplicates were automatically removed. Study titles and abstracts were screened for eligibility in Covidence by ND, and marked as “yes,” “no” or “maybe.” Queries (e.g., articles marked as “maybe”) were resolved by reviewing the full text. Any queries following full text screening were resolved through discussion and consensus with two additional team members (LC, RK). Full texts of articles that met inclusion criteria were uploaded to Covidence and independently reviewed by ND to confirm eligibility. Reasons for exclusions were recorded following a hierarchical tagging system in Covidence (not perinatal, not ART, not anxiety, ineligible study design). Full texts included in the systematic review were cross-checked a second time by ND prior to data extraction.

### 2.5 Data extraction

ND independently extracted data from all included articles using a template designed in Covidence following the PICO framework. Data extracted comprised: publication title, year, author(s), country, study design, sample size (ART group/SC group), mean age (ART group/SC group), exposure (types of ART), study inclusion and exclusion criteria, measure of anxiety symptoms, timing of measurement(s), results (prevalence or mean anxiety symptom score in ART and SC groups), statistical comparisons of differences in anxiety symptoms between groups, birth outcomes, and controls for confounding factors. A second reviewer, SB, independently double-coded all extracted data.

### 2.6 Assessment of risk of bias

Risk of bias within studies was formally assessed through the Effective Public Health Practice Project (EPHPP) quality assessment tool for quantitative studies (“^35^). ND employed the EPHPP tool to assess the risk of bias of all included studies. This entailed rating each study (where relevant) for selection bias, study design, confounders, masking, data collection methods, withdrawals and drop-outs, intervention integrity, and appropriateness of the conducted analyses. Following EPHPP guidelines, studies were assigned an overall rating of “strong” (no “high risk of bias” ratings), “moderate” (one “high risk of bias” rating), or “high risk of bias” (two or more “high risk of bias” ratings).

### 2.7 Approach to data synthesis

Due to the observational nature and varied risk of bias of included studies, a narrative synthesis was used to summarize the findings of the available evidence. Meta-analysis was not pursued due to methodological heterogeneity of included studies and concerns about bias when meta- analyzing observational studies (36). Further, included studies differed in ART type, gestational timing of assessment, anxiety symptom measure used, sample size, and confounder adjustment.

## 3 Results

### 3.1 Study selection

**Figure 1:**
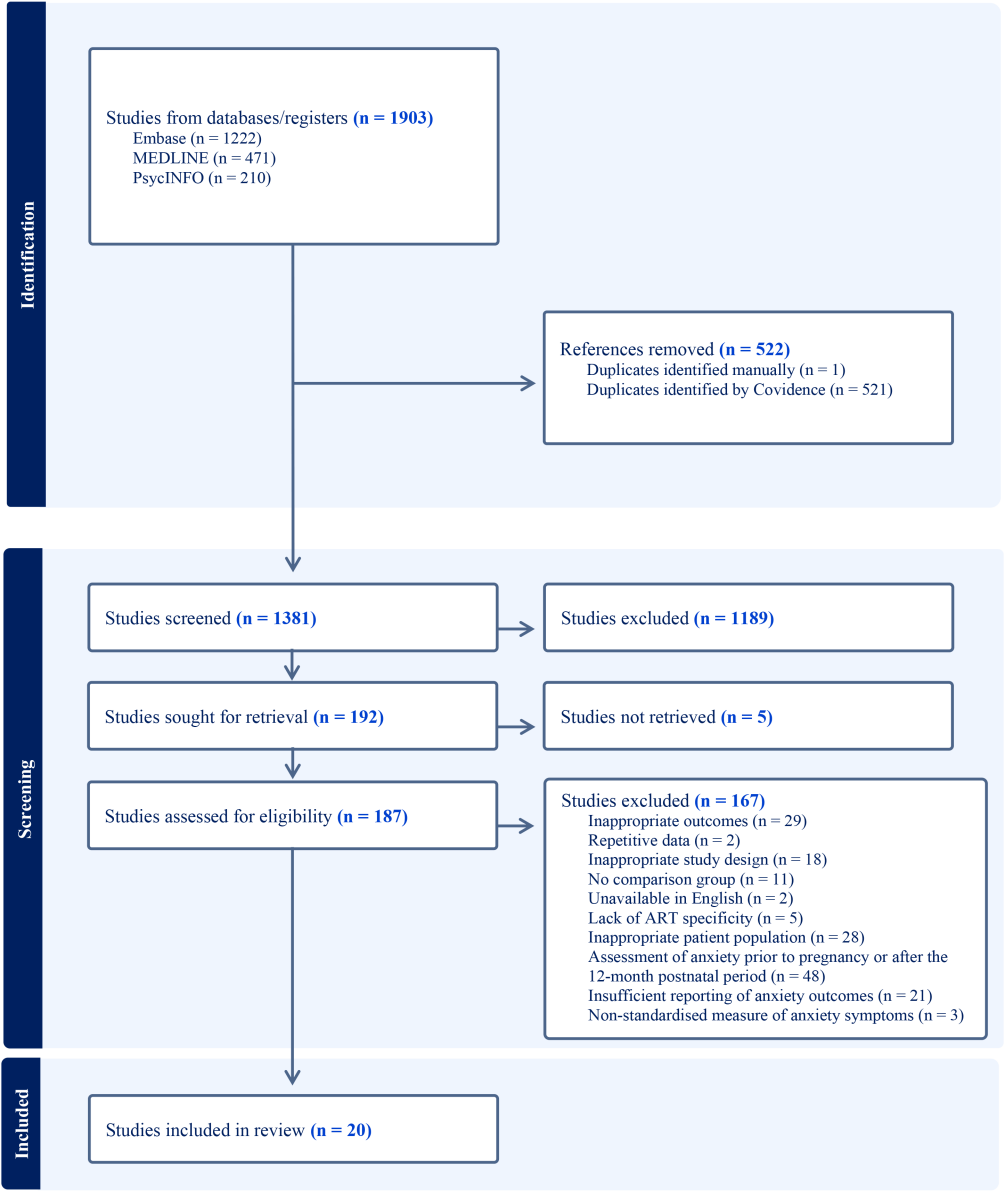
PRISMA flowchart of searches and selection. Adapted from Covidence.

The article selection process is presented in **Figure 1**. The systematic search of databases identified 1,903 studies, of which 522 duplicates were identified and removed. After title and abstract and full-text screening, 20 articles were included in this systematic review. The most common reason for exclusion was assessment of anxiety symptoms before pregnancy or after the 12-month postnatal period (n=48).

### 3.2 Characteristics of included studies

Study details and risk of bias scores are presented in **Table 1**. The primary findings are presented in **Table 2**, with anxiety symptom outcomes reported as prevalence (%) or mean scores, with statistical comparisons, where available. Included studies were predominantly conducted in European countries (n=10) and Australia (n=4), as well as the United States, Israel, Canada, Iran, India, and Russia. For articles that specified the date of data collection, were conducted between 1999 and 2021. Study settings included academic institutions, infertility treatment services, public and private hospitals, outpatient clinics, and community-based data collection sites. Included designs comprised cohort (n=15), case-control (n=1), and cross-sectional studies (n=4).

**Table 1:** Study characteristics.

Study	Data collection year	Country	Setting	Design	Type of ART	Sample size (ART / SC)	Mean age ± standard deviation (ART / SC)	Risk of bias
Fisher et al. (37)	N.S.	Australia	Academic institutions, infertility treatment services and public and private hospitals	Prospective cohort	ART	297 / 295	35.37 ± 4.62 / 32.05 ± 4.65	Moderate
Garcia-Blanco et al. (38)	2015	Spain	Hospital	Prospective cohort	IVF	60 / 183	33.63 ± 4.72 / 32.38 ± 4.22	High risk of bias
Harf-Kashdaei et al. (39)	N.S.	Israel	Outpatient clinics	Prospective controlled cohort	IVF	30 / 30	28.3 ± 2.45 / 28.5 ± 2.73	High risk of bias
Hjelmstedt et al. (40)	N.S.	Sweden	Hospital	Longitudinal cohort	IVF	57 / 43	32.3 ± 2.1 / 31.2 ± 1.8	Moderate
Hjelmstedt et al. (41)	N.S.	Sweden	Hospital	Longitudinal cohort	IVF	56 / 41	32.3 ± 2.1 / 31.3 ± 1.8	Moderate
Klock & Greenfeld (42)	N.S.	United States	Outpatient infertility and obstetrics practices	Prospective longitudinal cohort	IVF	74 / 40	33.7 ± 3.4 / 30.6 ± 4.4	Moderate
McMahon et al. (43)	N.S.	Australia	ART clinics and hospitals	Longitudinal cohort	ART	250 / 262	35.10 ± 4.28 / 32.20 ± 4.46	Moderate
McMahon et al. (44)	N.S.	Australia	Hospital	Prospective longitudinal cohort	IVF	70 / 63	34.6 ± 3.0 / 31.9 ± 2.4	High risk of bias
Pellerone et al. (45)	2020	Italy & Spain	Public health service childbirth preparation courses	Cross-sectional	ART	45 / 50	31.27 ± 4.88 / 29.94 ± 5.92	High risk of bias
Poikkeus et al. (46)	1999	Finland	Infertility clinics (Academic university, Family Federation of Finland, and a non-profit institute)	Prospective cohort	IVF, ICSI	367 / 379	33.0 ± 4.2 / 33.3 ± 3.0	High risk of bias
Raguz et al. (47)	2008-2010	Canada	Community-based data	Prospective cohort	Fertility drugs, AI, IUI, IVF, ICSI, embryo transfer	76 / 152	33.39 ± 5.11 / 31.28 ± 4.47	Moderate
Ranjbar et al. (48)	2019	Iran	Prenatal clinic	Longitudinal Cohort	ART	43 / 144	33.11 ± 4.39 / 31.81 ± 6.13	Moderate
Salevaara et al. (49)	N.S.	Finland	Academic university and fertility clinic	Nested case-control	Oocyte donation, IVF/ICSI	26 (Oocyte donation); 52 (IVF/ICSI) / 52	36.1 ± 6.1 (Oocyte donation); 35.2 ± 4.5 (IVF/ICSI)/ 35.1 ± 4.3	Moderate
SalihJoelsson et al. (50)	2012-2015	Sweden	Fertility and antenatal clinic	Cross-sectional	IVF, OI, IUI, sperm donation, ovum donation	143 / 2972	32.3 ± 4.9 / 29.3 ± 4.8	High risk of bias
Saravanan et al. (51)	2020-2021	India	Private obstetrics and gynecology hospital	Prospective longitudinal cohort	ART	44 / 47	32.72 ± 2.05 / 31.46 ± 1.86	Moderate
Simoni et al. (52)	2005-2009	United States	Obstetric practices	Prospective cohort	ART (including autologous gametes and donor gametes)	191 / 1466	35.0 ± 4.2 / 31.8 ± 4.7	Moderate
Szemes et al. (53)	2012-2013	Hungary	Hospital department of obstetrics and gynecology	Cross-sectional	OI, IUI, IVF	100 / 885	35.2 (± N.S.)/ 32.6 (± N.S.)	High risk of bias
Tendais & Figueiredo (54)	N.S.	Portugal	Public hospitals	Prospective longitudinal Cohort	ICSI, IVF, OI, AI, surgery	17 (Singletons); 19 (Twins) / 203 (Singletons); 28 (Twins)	N.S.	Moderate
Vilska et al. (55)	1999	Finland	Infertility clinics (Academic university, Family Federation of Finland, and a non-profit institute)	Prospective longitudinal cohort	ART	367 (Singletons); 91 (Twins) / 379 (Singletons); 20 (Twins)	33.0 ± 4.2 (Singletons); 31.7 ± 4.0 (Twins) / 33.3 ± 3.0 (Singletons); 33.1 ± 5.3 (Twins)	Moderate
Yakupova et al. (10)	N.S.	Russia	Scientific Center for Obstetrics, Gynecology, and Perinatology	Cross-sectional	IVF	62 / 162	33.2 ± 5.2 / 30.8 ± 5.3	High risk of bias

SC, Spontaneous conception; N.S., Not specified; ART, Assisted Reproductive Technology; IVF, In Vitro Fertilization; ICSI, Intracytoplasmic sperm injection; AI, Artificial insemination; OI, Ovulation induction; IUI, Intrauterine insemination; IVF-ET, In vitro fertilization with embryo transfer..

**Table 2:** Anxiety symptoms measurement and outcomes.

Study	Anxiety scale	Timing of measurement	ART group results (Mean ± standard deviation OR prevalence)	SC group results (Mean ± standard deviation OR prevalence)	Significant differences
Fisher et al. (37)	STAI	Third trimester	SA: 30.5 ±7.4 TA: 33.0 ± 8.1	SA: 32.7 ± 9.6 TA: 34.7 ± 8.6	ART women had sig. lower SA (p < 0.05) and TA (p < 0.05) scores than SC.
Garcia-Blanco et al. (38)	STAI	T1: Third trimester T2: 48 hours after birth T3:3 months postnatal	T1: 22.22 ± 9.57 T2: 17.69 ± 8.57 T3: 17.58 ± 10.18	T1: 17.27 ± 10.09 T2: 15.57 ± 10.28 T3: 14.70 ± 10.46	IVF mothers had sig. higher SA scores at T1 (p = 0.016); both groups declined in parallel with no sig. difference in rate of change from T1 to T2 (p = 0.37) or T2 to T3 (p = 0.36).
Harf-Kashdaei et al. (39)	STAI	Third trimester	TA: 33.82 ± 7.81 SA: 34.83 ± 9.92	TA: 36.76 ± 9.99 SA: 30.64 ± 9.87	No sig. group differences in SA (p > 0.20) or TA (p > 0.20). 10% of the women in the control group scored higher than the clinical anxiety cutoff score (51) versus 0% in the IVF group.
Hjelmstedt et al. (40)	ERPS	T1: Gestational week 13 T2: Gestational week 26 T3: Gestational week 36	Pregnancy loss anxiety (scored 2 – 10)*: T1: ~7.1 T2: ~5.7 T3: ~5.7 Baby health anxiety (scored 1 to 5)*: T1: ~3.5 T2: ~3.7 T3: ~3.9	Pregnancy loss anxiety (scored 2 – 10)*: T1: ~5.0 T2: ~4.7 T3: ~4.6 Baby health anxiety (scored 1 to 5)*: T1: ~4.1 T2: ~4.2 T3: ~3.9	Overall pregnancy loss anxiety was higher in IVF women (*p* < 0.001). Reduction in pregnancy loss anxiety was more pronounced among IVF women from 13 to 36 weeks (*p* < 0.01). There was an increase from pregnancy weeks 13 to 36 (paired t = -2.77, *p* < 0.01) in the IVF group, whereas SC showed no change (paired t= 0.64, N.R.).
Hjelmstedt et al. (41)	STAI	T1: Gestational week 13 T2: Gestational week 26 T3: Gestational week 36	TA T1: 33.1 ± 6.7 SA T2: 29.8 ± 6.2 T3: 31.0 ± 6.8	TA T1: 30.9 ± 6.3 SA T2: 30.3 ± 6.8 T3: 28.9 ± 4.8	Statistical comparison N.R.
Klock & Greenfeld (42)	STAI	T1: Gestational week 12 T2: Gestational week 28	T1: SA: 35.33 ± 11.84 TA: 32.91 ± 9.19 T2: SA: 32.00 ± 10.01 TA: 31.41 ± 8.52	T1: SA: 32.33 ±12.02 TA: 33.20 ± 10.82 T2: SA: 31.03 ± 13.27 TA: 32.09 ± 11.11	No sig. group differences (p N.R.).
McMahon et al. (43)	STAI; PFA	T1: Third trimester T2: 4 months postnatal	SA: T1: 30.49 ± 7.39 T2: 30.80 ± 8.50 TA: T1: 32.83 ± 8.02 T2: N.R. PFA: T1: 19.27 ± 6.35	SA: **T1:** 32.52 ± 9.62 **T2:** 31.06 ± 8.42 TA: **T1**: 34.51 ± 8.55 **T2:** N.R. PFA: **T1:** 17.91 ± 6.08	Sig. lower SA (p < .01) and TA (p < .05), but higher pregnancy-focused anxiety (*p* < .05) in ART group during the third trimester. Post-partum SA difference n.s..
McMahon et al. (44)	STAI; Pregnancy-specific anxiety measures	~30 gestational weeks (range 28–33)	SA: 32.23 ± 10.62 TA: 34.72 ± 9.32 Pregnancy-specific anxiety: N.R.	SA: 30.12 ± 6.67 TA: 35.55 ± 7.00 Pregnancy-specific anxiety: N.R.	N.s. differences in TA or SA scores (p N.R; p = 0.07). Sig. group differences were found in anxiety concerning health and defects in the child (p=0.008), trust in pregnancy survival (p<0.001), delay in telling others about pregnancy (p=0.034), threats to the child during birth (p<0.001), and negative feelings about childbirth (p=0.003). N.s. group differences were observed for tolerance for medical interventions during birth (p=0.078) and anxiety about postnatal separation (p=0.076).
Pellerone et al. (45)	STAI	Gestational weeks 23 - 37	SA: 43.27 ± 9.62 TA: 44.47 ± 7.55	SA: 39.28 ± 9.24 TA: 41.94 ± 7.92	Sig. higher SA in ART group (p < 0.05). No sig. difference in TA (p > 0.05).
Poikkeus et al. (46)	Fear-of-Childbirth Questionnaire (revised); Pregnancy Anxiety Scale	Gestational week 20 ± 3.2	Severe Fear of Childbirth: 11.4%; Severe Pregnancy-Related Anxiety: 12.5%	Severe Fear of Childbirth: 10.6%; Severe Pregnancy-Related Anxiety: 10.0%	No sig. group differences in severe fear of childbirth (p=0.56) or severe pregnancy-related anxiety (p=0.16).
Raguz et al. (47)	STAI	4 months postnatal	High anxiety (prevalence): 8.1%	High anxiety (prevalence): 16.9%	No sig. group differences (p=0.08).
Ranjbar et al. (48)	PRAQ-17	T1: Gestational week 12 T2: Gestational week 36	T1: 51.9 ± 18.2 T2: 33.67 ± 11.68	T1: 51.36 ± 9.59 T2: 32.52 ± 13.36	No sig. group differences at T1 (p=0.87) or T2 (p=0.61). Scores declined in both groups from T1 to T2 but were not significantly different (p=0.84)..
Sälevaara et al. (49)	GHQ-36	T1: Gestational weeks 18-20 T2: 2 months postnatal T3: 12 months postnatal	Oocyte donation: T1: 1.33 ± 0.32 T2: 1.28 ± 0.22 T3: 1.24 ± 0.19 IVF/ICSI: T1: 1.38 ± 0.34 T2: 1.50 ± 0.49 T3: 1.44 ± 0.55	T1: 1.52 ± 0.41 T2: 1.63 ± 0.56 T3: 1.56 ± 0.48	T1: No sig. group differences (p N.R.) T2: Oocyte donation vs SC: Oocyte donation mothers reported sig. lower anxiety (p = 0.02) IVF/ICSI vs SC: No significant difference (p > 0.05) T3: Oocyte donation vs SC: Oocyte donation mothers reported sig. lower anxiety (p = 0.01) IVF/ICSI vs SC: No significant difference (p > 0.05)
SalihJoelsson et al. (50)	HADS-A≥8	During pregnancy	HADS-A ≥ 8 (prevalence): 21.1%	HADS-A ≥ 8 (prevalence): 18.8%	No sig. group differences (p=0.20)
Saravanan et al. (51)	GAD-7	T1: 7 months gestation T2: 2 weeks postnatal	T1: 5.98 ± 2.12 T2: 5.56 ± 1.88	T1: 3.22 ± 1.60 T2: 4.00 ± 1.39	ART group had sig. higher anxiety scores at 7 months gestation (p < 0.001) and 2 weeks postnatal (p < 0.001)
Simoni et al. (52)	EPDS-3A	T1: <17 weeks gestation T2: 28 (±2) weeks gestation T3: 8 (±4) weeks postnatal	Reported medians (IQR) T1: 3.3 (0.0–13.3) T2: 3.3 (0.0–10.0) T3: 3.3 (0.0–10.0)	Reported medians (IQR) T1: 6.7 (0.0–13.3) T2: 3.3 (0.0–10.0) T3: 3.3 (0.0–10.0)	No sig. group differences in median anxiety (all ps >0.304). No sig. differences in the prevalence of anxiety (EPDS-3A ≥ 10) at each interview (all ps>0.28).
Szemes et al. (53)	STAI	22 - 40 weeks gestation	Clinical-level anxiety (≥ 50 points) (prevalence): 9%, Average anxiety score: 34.0 (± N.R.)	Clinical-level anxiety (≥ 50 points) (prevalence): 11.6% Average anxiety score: 35.6 (± N.R.)	No sig. group differences (p > 0.05).
Tendais & Figueiredo (54)	STAI	T1: 8–15 weeks gestation T2: 20–24 weeks gestation T3: 28–34 weeks gestation T4: During hospital stay after childbirth T5: 3 months postnatal	Singletons: T1: 33.9 ± 1.59 T2: 30.0 ± 1.69 T3: 31.6 ± 1.87 T4: 41.8 ± 2.11 T5: 28.3 ± 1.65 Twins: T1: 37.0 ± 1.52 T2: 34.8 ± 1.58 T3: 36.5 ± 1.72 T4: 39.8 ± 2.14 T5: 36.0 ± 1.71	Singletons: T1: 34.6 ± 0.47 T2: 33.1 ± 0.49 T3: 34.9 ± 0.53 T4: 34.1 ± 0.64 T5: 30.0 ± 0.52 Twins: T1: 34.2 ± 1.28 T2: 31.8 ± 1.27 T3: 33.9 ± 1.43 T4: 36.1 ± 1.77 T5: 32.0 ± 1.37	No sig. differences between ART and SC mothers of twins (p > 0.05). ART mothers had sig. higher anxiety scores during hospital stay after childbirth compared to SC mothers (p < 0.001). Significant increase in anxiety from pregnancy to post-partum for ART mothers of singletons (p < 0.001).
Vilska et al. (55)	GHQ-36 11-item anxiety subscale	T1: 2nd trimester T2: 2 months postnatal T3: 12 months postnatal	Singletons: T1: 1.42 ± 0.02 T2: 1.45 ± 0.02 T3: 1.43 ± 0.03 Twins: T1: 1.53 ± 0.04 T2: 1.60 ± 0.05 T3: 1.56 ± 0.06	Singletons: T1: 1.52 ± 0.02 T2: 1.47 ± 0.03 T3: 1.47 ± 0.03 Twins: T1: 1.50 ± 0.09 T2: 1.67 ± 0.11 T3: 1.87 ± 0.14	ART and SC mothers of twins had more anxiety symptoms than mothers of singletons (p < 0.05) at 2 months postnatal. SC mothers of twins had more anxiety symptoms than ART mothers of twins and singletons (p < 0.01 for parenthood and p < 0.05 for group) at 12 months postnatal.
Yakupova et al. (10)	EPQ-A	Second & third trimesters	High anxiety level (prevalence): 4.8%	High anxiety level (prevalence): 4.3%	No sig. group differences (p N.R.)

* All approximate values were pulled from graphs and given as best estimates.

SC, Spontaneous conception; STAI, State Trait Anxiety Inventory; ERPS, Emotional Responses to Pregnancy Scale; PRAQ-17, van Den Bergh Pregnancy-Related Anxiety Questionnaire; HADS-A≥8, Anxiety subscale of the Hospital Anxiety and Depression Scale; EPDS-3A, Edinburgh Postnatal Depression Scale anxiety subscale; GAD-7, Generalized Anxiety Disorder-7 Scale; GHQ-36, 36-item version of the General Health Questionnaire; SA, State anxiety; TA, Trait anxiety; Pregnancy-Focused Anxiety, PFA; Eysenck Personality Questionnaire-Anxiety Scale, EPQ; N.R., Not reported.

All types of ART were received by participants in included studies: IVF, frozen embryo transfer, intracytoplasmic sperm injection (ICSI), artificial insemination (AI), and ovulation induction (OI). Seven studies used IVF exclusively, while the majority either grouped all ART types together or did not disaggregate findings by treatment modality, precluding systematic comparison across ART methods. Sample sizes varied, with ART groups ranging from 30 to 367 participants and SC groups ranging from 30 to 2,972 participants. Across studies, the mean age of ART groups ranged from 28.3 to 36.1 years and 28.5 to 35.1 years for SC groups. One study analyzed ART in separate sub-groups (oocyte donation and IVF/ICSI with own gametes) (49), and two studies analyzed singleton and twin pregnancies in separate subgroups for both ART and SC groups (54, 55).

### 3.3 Risk of bias assessment

Of the 20 included studies, none was rated “strong” on the EPHPP scale, due to a lack of randomization in observational studies. The majority (n=12) was rated “moderate,” and eight studies were rated as being at “high risk of bias.” Most studies were rated as being at high risk of bias for adequate masking, with assessors often not masked, participants aware of the research objectives, or this information not being reported. Selection bias was also prevalent, with 17 studies using non-random sampling and inclusion criteria that limited generalizability (e.g., sampling only nulliparous women). Additionally, several studies reported low participation rates or did not report these. Twelve studies statistically adjusted for or controlled by design for at least one confounding factor, with maternal age (n=9; six via covariate adjustment, three via matched design) and parity/plurality (n=8; four via covariate adjustment, two via matched design, and two via stratified analysis) the most common. Three additional studies restricted their samples to control for age and/or plurality (40, 41, 47).

Other covariates included pregnancy complications, gestational age at assessment, socioeconomic indicators (e.g., education, occupation), and lifestyle behaviors (e.g., smoking). Lastly, masking and concerns of high drop-out rates in eight studies contributed to the elevated risk of bias.

### 3.4 Anxiety symptom assessments and timing

Included studies used a range of validated measures to assess anxiety symptoms. The State and Trait Anxiety Inventory (STAI) (56) was the most frequently used measure (n=11). Other studies assessed generalized anxiety symptoms using the General Health Questionnaire (Full-scale GHQ-36 and GHQ-36 11-item subscale) (n=2), Hospital Anxiety and Depression Scale (HADS-A≥8) (n=1), Edinburgh Postnatal Depression Scale anxiety subscale (EPDS-3A) (n=1), Generalized Anxiety Disorder-7 Scale (GAD-7) (n=1), and the Eysenck Personality Questionnaire -Anxiety Scale (EPQ-A) (n=1). Pregnancy-specific measures included the Emotional Responses to Pregnancy Scale (ERPS) (n=1), van Den Bergh Pregnancy-Related Anxiety Questionnaire (PRAQ-17) (n=1), Pregnancy Anxiety Scale (PAS) (n=1), Pregnancy-Focused Anxiety (PFA) (n=1), and the revised Fear-of-Childbirth Questionnaire (n=1).

Anxiety symptom measurements were predominantly conducted during pregnancy (n=19), with seven further studies administering assessments during both pregnancy and postpartum. Only one study administered an anxiety assessment solely in the postnatal period (47).

### 3.5 Anxiety symptoms during pregnancy

#### 3.5.1 First trimester *(weeks 1 to 12)*

Two studies reported generalised anxiety symptoms during the first trimester and one used a pregnancy-specific scale. At gestational week 12, there were no significant differences in anxiety symptoms between ART and SC groups on the PRAQ-17 (48) or the STAI (42). In assessments between gestational weeks 8 to 15, Tendais & Figueiredo (54) likewise reported no significant differences (*p*>0.05) between groups on the STAI. Together, these findings do not provide evidence of differences in first trimester anxiety symptoms between women who conceive via ART and SC.

#### 3.5.2 Second trimester *(weeks 13 to 27)*

Eight studies assessed anxiety symptoms during the second trimester. Notably, at 13 weeks, Hjelmstedt et al. (40) administered the pregnancy-specific ERPS and found that women who conceived following IVF reported higher pregnancy-loss anxiety than women who conceived by SC (*p* < 0.001). Yet, a follow-up study by Hjelmstedt and colleagues (41) reported STAI scores at 13 weeks’ gestation but did not report statistical comparisons; mean trait-anxiety (TA) and state-anxiety (SA) differences between groups appeared to be minimal.

All remaining second trimester findings did not report significant differences in anxiety symptoms between groups across both generalized and pregnancy-specific measures in the second trimester (10, 46, 49, 52, 54, 55). Using the EPDS-3A prior to 17 weeks’ gestation, Simoni et al. (52) found no significant ART versus SC group differences in median anxiety scores (*p >*0.304) or symptom prevalence (EPDS-3A ≥ 10; *p*>0.28). Three studies assessed symptoms around 20 –weeks’ gestation using pregnancy-specific measures (the Fear-of-Childbirth Questionnaire (revised) and PAS) and generalized anxiety measures (GHQ-36 and STAI) (46, 49, 54). Findings supported no significant differences between groups in severe fear of childbirth (*p* = 0.56) or pregnancy-related anxiety (*p* = 0.16) (46). Likewise, at 18 to 20 weeks, Sälevaara et al. (49) reported non-significant differences (p-value not reported) on the GHQ-36 for both oocyte donation and IVF/ICSI compared to SC women. Further, at 20 to 24 weeks, Tendais & Figueiredo (54) found no significant differences in STAI scores between ART and SC women who were expecting twins (*p* > 0.05). Hjelmstedt et al. (41) also reported mean symptoms at 26 weeks’ gestation but did not include statistical comparisons between groups. Lastly, Vilska et al. (55) and Yakupova et al. (10) assessed symptoms throughout the second trimester and did not report significant differences between ART and SC women with both singleton and twin pregnancies on the EPQ-A.

#### 3.5.3 Third trimester *(week 28 to delivery)*

The majority (n=16) of included studies assessed anxiety symptoms during the third trimester, reporting mixed results that varied by both direction and type of anxiety measure used. Five third trimester evaluations using the STAI (at 22-40, 28, 28-34, and 36 weeks and throughout the third trimester) reported no significant group differences (39, 42, 48, 53, 54). Studies using the EPDS-3A, HADS-A, and EPQ-A reported no significant differences between ART and SC groups (50, 52). Yakupova et al. (10) similarly reported comparable distributions on the EPQ-A, though without formal statistical testing.

A series of findings reported heightened anxiety symptoms in the ART group compared to the SC group (n=5). Pellerone et al. (45) assessed the STAI at 23 to 37 weeks and found significantly higher state anxiety in the ART group compared to the SC group (ART: M = 43.27, SD = 9.62; SC: M = 39.28, SD = 9.24; *p* < 0.05), but no difference in TA (*p*>0.05). Notably, the ART group SA mean score was above the conventional clinical threshold. Similarly, Garcia-Blanco et al. (38) reported significantly higher state anxiety scores at 32 weeks’ gestation in women who conceived through IVF compared to women who conceived without assistance (ART: M = 22.22, SD = 9.57; SC: M = 17.27, SD = 10.09; p=0.016). The IVF group mean exceeded the clinical threshold for this version of the STAI (cut-off ≥19), unlike the SC group mean, indicating the statistically significant difference corresponded to clinically elevated scores in the IVF group. Saravanan et al. (51) found significantly higher GAD-7 scores in the ART group at 7 months’ gestation compared to the SC group (ART: M = 5.98, SD = 2.12; SC: M = 3.22, SD = 1.60; p<0.001). The ART group mean fell in the mild anxiety range (GAD-7 ≥5), whereas the SC group mean indicated minimal symptoms. McMahon et al. (44) reported non-significant differences between ART and SC groups on SA and TA at 30 weeks’ gestation (ART: SA M = 32.23 ± 10.62, TA M = 34.72 ± 9.32; SC: SA M = 30.12 ± 6.67, TA M = 35.55 ± 7.00; p-value not reported; p = 0.07 respectively). However, women who conceived via ART reported significantly higher pregnancy-focused anxiety, particularly regarding concerns about the health (*p* = 0.008) and survival of the unborn (*p* < 0.001). Although Hjelmstedt et al. (40) found that pregnancy loss anxiety decreased more among IVF women as their pregnancies progressed from 13 to 36 weeks (*p* < 0.01), scores remained elevated relative to SC women at 36 weeks’ gestation (overall *p* < 0.001). Additionally, women who had conceived through IVF showed increased anxiety about the health of their baby from weeks 13 to 36 (paired t= -2.77, *p* < 0.01) unlike SC women (paired t=0.64, not significant, p-value not reported).

Conversely, two studies reported lower anxiety symptoms in the ART group relative to the SC group on the STAI (37, 43). Fisher et al. (37) found that ART women exhibited significantly lower SA and TA scores (ART: SA M = 30.5 ± 7.4, TA M = 33.0 ± 8.1; SC: SA M = 32.7 ± 9.6, TA M = 34.7 ± 8.6; *p* < 0.05) than those in the SC group at around 32 weeks’ gestation. Further, scores for both groups fell below the STAI clinical threshold. Likewise, McMahon et al. (43) reported that the ART group exhibited significantly lower levels of both SA and TA (ART: SA M = 30.49 ± 7.39, TA M = 32.83 ± 8.02; SC: SA M = 32.52 ± 9.62, TA M = 34.51 ± 8.55; *p* < 0.05) but higher pregnancy-focused anxiety scores compared to their SC group (ART: PFA M = 19.27 ± 6.35; SC: PFA M = 17.91 ± 6.08; *p* < 0.05) at around 32 weeks’ gestation. While Harf-Kashdaei et al. (39) found no significant differences in SA and TA between groups on the STAI at around an average of 30 to 31 weeks, 10% of SC women (n=30) but 0% of ART women (n=30) scored above the clinical anxiety cut-off (score of 51).

### 3.6 Postnatal anxiety symptom outcomes

Findings for differences between ART and SC women postpartum varied across studies. Notably, all postnatal assessments utilized generalized anxiety rather than pregnancy-specific measures. Two findings reported significantly heightened anxiety symptoms in ART compared to SC mothers during the first postnatal year. Tendais & Figueirido (54), using the STAI-S, reported that women who delivered singleton babies following ART had significantly higher anxiety scores during their immediate postnatal ward stay, compared with women who conceived by SC (ART singletons: M = 41.8, SD = 2.11; SC singletons: M = 34.1, SD = 0.64;*p* < 0.001). The ART singletons mean of 41.8 exceeded the conventional STAI-S clinical threshold of 40. They also identified a significant increase in mean anxiety symptoms between late pregnancy (28–34 weeks gestation; ART singletons M = 31.6) and the immediate postpartum hospital stay (ART singletons M = 41.8; *p* < 0.001). Saravanan et al. (51) found that women in the ART group exhibited significantly higher GAD-7 scores than SC women, two weeks postpartum (ART: M = 5.56, SD = 1.88; SC: M = 4.00, SD = 1.39; *p* < 0.001).

Two studies reported lower anxiety scores in the ART group than the SC group, postpartum. Vilska et al. (55), utilizing the 11-item GHQ-36 anxiety subscale, reported higher anxiety symptoms among women who conceived twins by SC than among women who conceived both twins and single babies via ART at 12 months postpartum (SC twins: M = 1.87 ± 0.14; ART twins: M = 1.56 ± 0.06; ART singletons: M = 1.43 ± 0.03; *p* < 0.01 for parenthood and *p* < 0.05 for group). Sälevaara et al. (49) used the full-scale GHQ-36 to compare three groups of women who conceived via oocyte donation, IVF/ICSI, and SC in a stratified analysis. Women who conceived via oocyte donation had significantly lower anxiety scores than SC mothers at two months (oocyte donation: M = 1.28 ± 0.22; SC: M = 1.63 ± 0.56; *p* = 0.02) and 12 months (oocyte donation: M = 1.24 ± 0.19; SC: M = 1.56 ± 0.48; *p* = 0.01) postpartum. This finding did not apply to the IVF/ICSI group (2 months: IVF/ICSI: M = 1.50 ± 0.49; 12 months: M = 1.44 ± 0.55), which showed no significant differences from the SC group at either time point (*p*>0.05).

Finally, four further studies reported no significant postnatal group differences. At three months postpartum, no significant STAI differences were observed among mothers of twins in the ART and SC groups, whereas women of singletons had endorsed an increase in anxiety symptoms immediately after birth that resolved by three months (54). Garcia-Blanco et al. (38) found that gestational group differences in SA were no longer statistically significant at 48 hours and three months postnatal. A study that used the EPDS-3A (measured between 4 and 12 weeks postpartum) did not find significant group differences (52). Likewise, a study that used the GHQ-36 (measured at 2 and 12 months postpartum) also did not find significant group differences (49). Lastly, two studies using the STAI reported no significant differences between ART and SC groups at four months postpartum (43, 47).

## 4 Discussion

### 4.1 Summary of evidence

The present study is the first systematic review to compare anxiety symptoms between women who conceived following ART and through SC throughout pregnancy and until 12 months postpartum. We contribute to a mixed literature surrounding maternal mental health outcomes following ART. Across 20 studies, findings varied regarding differences in perinatal anxiety symptoms between women who conceived via ART versus SC. About half of the included studies reported significant differences at one or more time points. In the first and second trimesters, all but one study reported non-significant differences between ART and SC groups, irrespective of the specific gestational time point, though the small sample sizes in several early-pregnancy studies limit confidence in these null findings. One study reported higher pregnancy loss anxiety symptoms in IVF pregnancies at 13 weeks’ gestation (40).

Conversely, during the third trimester, findings were more mixed. Some studies identified elevated pregnancy-specific anxiety symptoms in ART women (e.g., regarding pregnancy loss and childbirth), while others found lower SA and TA symptoms in the ART group, or non-significant differences. Differences were more often significant when pregnancy-specific scales (e.g., PRAQ-17, ERPS) were employed. Studies that used the STAI scale generally did not identify higher anxiety symptoms in the ART group. Notably, this pattern suggests a relative vulnerability in ART women to pregnancy-specific, rather than generalized, anxiety symptoms, which may suggest heightened concerns around pregnancy loss and fetal wellbeing after ART.

The lack of elevated symptoms in early pregnancy for IVF women may indicate that anxiety at this stage is more strongly linked to concerns about pregnancy viability than to the number of fetuses. While Vilska et al. (55) found that women who had given birth to twins had higher anxiety symptoms than women who had singleton pregnancies, regardless of ART or SC, such plurality-related concerns may not yet be prominent in early pregnancy. This suggests that stressors and other factors associated with having twins are more strongly associated with postnatal anxiety symptoms than the conception method. Given that ART increases the likelihood of multiple births (19, 57), plurality may act as a confounder, inflating observed group differences in studies that did not measure or control for multiple pregnancies, or alternatively as part of the causal pathway linking ART to anxiety outcomes. However, Garcia-Blanco et al. (38) observed elevated anxiety symptoms among women who received ART after adjusting for multiple pregnancies. Further, null findings in early pregnancy may contradict the expectation that women who have experienced infertility worry more about pregnancy viability and potential loss. Additionally, maternal age is the predominant factor linked to miscarriage rates, regardless of conception method, and thus may underlie early pregnancy anxiety symptoms (58). While maternal age was the most frequently controlled variable across included studies (n=11), findings from studies that did adjust for age were not consistently different from those that did not. Specifically, six studies that adjusted for maternal age still reported significant differences, suggesting maternal age alone does not fully account for observed group differences. The cumulative burden of ART-related distress may continue into pregnancy and culminate in heightened vigilance toward pregnancy-specific threats.

Postpartum anxiety symptoms varied across instruments used, and assessments were conducted less frequently over the 12-month period compared to during pregnancy. Two studies (51, 54) found higher postpartum anxiety symptoms, while two studies reported lower postpartum anxiety symptoms in specific ART subgroups (women who received oocyte-donation 2 (49) and conceived twins via ART (55), relative to SC. However, these findings may be influenced by factors that make oocyte donation or dual embryo transfer more likely, such as maternal age. These findings demonstrate variation in postnatal anxiety following ART (54). Four other studies reported non-significant differences postpartum (38, 43, 47, 52). These findings illuminate women’s experiences throughout their ART experience, but there is a lack of longitudinal studies measuring women’s antenatal anxiety symptoms and later in motherhood.

The variability in postpartum findings may be due to heterogeneity of assessment timing and measures. Specifically, postpartum assessments range from the immediate hospital stay to twelve months postnatal and used a range of assessments both differing in psychometric properties and the time frames questioned. In addition to measurement heterogeneity, other psychosocial and environmental factors that influence women’s mental health in the first postnatal year likely play a role with ART-related and postnatal experiences at large. Specifically, recent reviews have highlighted psychosocial correlates such as the role of partner support (59), perceived and received social support (e.g., friends, family) (60), pay and length of parental leave (61), intimate partner violence around pregnancy (62), and socioeconomic determinants (e.g., household food insecurity) (63). Emerging attention has also been placed on environmental exposures such as ambient air pollution and green space (64, 65). Additionally, anxiety associated with ART may diminish over time or interact with other stressors, such as birth trauma and infant health, which were infrequently recorded by the included studies.

### 4.2 Limitations

No studies were rated “strong” on risk of bias assessment due to the observational nature of studies, indicating the need for caution when interpreting findings. Selection bias was widespread, limiting generalizability to broader populations. Studies varied in their reporting of demographic information, complicating the depth of data extraction and comparison between studies. Age was reported across studies, but just over half reported maternal education or an alternative socioeconomic metric (e.g., household income), and fewer reported racial/ethnic demographic information for their sample. Limited reporting of participant demographics limited the ability to compare how different ethnic and socio-economic groups may experience ART. Most included studies were conducted in high-income countries (n=18) and did not report whether ART received was state, health insurance, or self-funded. Only one study included private health insurance as a covariate in analyses (37). Most studies did not control for baseline anxiety symptoms or infertility distress, both of which may impact anxiety symptoms. Additionally, the number of prior ART cycles undertaken was infrequently accounted for, which may influence subsequent anxiety symptoms. Further, preconception anxiety symptom data fell outside the scope of this review; thus, there remains uncertainty surrounding whether observed group differences reflect the impact of ART on anxiety or pre-existing differences between women who underwent fertility treatment compared to those who conceived spontaneously. Publication bias was not formally assessed, which would have necessitated a meta-analytic framework. Yet, null findings in the literature may be underestimated, and thus should be considered when interpreting ART and SC group differences.

All included studies reported anxiety symptoms on validated scales rather than clinical diagnostic measures, so findings may not accurately reflect the prevalence or severity of anxiety disorders. Use of a range of symptom measurement tools across studies introduced heterogeneity and highlighted the limited use of validated scales for perinatal anxiety symptoms, contributing to likely underreporting. Several studies also had small sample sizes, and thus null findings should be interpreted with caution because there may have been insufficient power to detect effects. Additionally, two studies reported group differences without formal statistical testing. Our study was limited by single-reviewer title and abstract screening, and risk of bias assessment, which we partly mitigated by ND double screening titles and abstracts.

### 4.3 Future directions

Future research should consider a wider range of potential factors contributing to post-ART anxiety symptoms, such as financial security (e.g., funding support and the financial burden of treatment) and support networks (e.g., partner, family, and peer involvement). While these factors are often measured in non-perinatal studies of women’s mental health, they remain overlooked in the ART literature. Additionally, certain minoritized groups, including LGBTQ+ individuals, who may disproportionately access ART, may face specific stressors and barriers related to their fertility experience, which impact their risk of perinatal anxiety symptoms (6). ART type, particularly more invasive versus less invasive methodologies, also warrant further examination in their roles in distinct anxiety profiles.

The nature of national register-based cohort data sets enables comprehensive, longitudinal evaluation of mechanistic hypotheses. National registers have identified associations between ART and mental health conditions, such as adjustment disorders and depression (66, 67). While our review focused on anxiety symptoms, more extensive research has been conducted surrounding perinatal depression symptoms, particularly extending into the postnatal period (29). Depression symptoms may have differing trajectories or pathways to anxiety symptoms and warrant further consideration, both in co-occurring and individual symptom pathways. Current perinatal depression cohort studies can be used to highlight the value of large-scale data in uncovering such symptom pathways. For example, recent findings in a Danish cohort revealed a 13% lower adjusted risk of postpartum depression in the medically assisted reproduction (MAR) group compared to SC (68). Alternatively, a US cohort revealed that ART exposure was not associated with PPD when pregnancy intention was included in analyses (69). Despite these findings, few studies specifically track postnatal symptomatology at multiple timepoints, and the inclusion of such repeated assessments has the potential to inform women’s longitudinal trajectories after ART. Future work should prioritize prospective longitudinal cohort studies beginning at the point of fertility treatment seeking, prior to conception, to establish baseline symptomatology and thus better map subsequent anxiety symptom trajectories.

Randomized controlled trials (RCTs) have evaluated interventions to reduce anxiety symptoms during the early stages of ART, such as acupuncture (70), mindfulness (71), virtual reality (72). For example, a multisite Australian RCT in women (n= 848) undergoing a fresh IVF cycle examined anxiety symptoms at baseline, embryo transfer, and 14 weeks post-enrolment (70). Participants in the acupuncture group had lower anxiety symptoms following embryo transfer than participants who received sham acupuncture. The authors identified declines in emotional well-being (measured by the Emotional Well−Being sub−scale of the MOS 36−Item Short Form Health Survey) three months post-IVF cycle, indicating ongoing unmet needs. Yet, intervention studies do not report longer-term anxiety outcomes later in pregnancy or postpartum and more extended follow-up is needed.

A handful (n=2) of included studies also measured non-birthing partner anxiety symptoms, which were not synthesized within the scope of this review. Findings reported that ART fathers exhibited higher worry about pregnancy loss and their baby’s safety compared to control men (40) and did not endorse an association between mode of conception and paternal anxiety symptomatology (49). Future systematic reviews should explore partner ART experiences and examine their potential impacts on the birthing partner and child. Lastly, given the specificity of women’s individual fertility, conception, birth, and motherhood experiences, qualitative research is especially needed to explore women’s experiences of perinatal anxiety in depth. A recent systematic review by Maehara and colleagues (73) explored the limited qualitative research (n=7) on the transition to motherhood in ART samples, emphasizing the need for support to address heightened anxiety and unique challenges such as delayed fetal attachment, maternal identity formation, and self-perceptions of being infertile.

### 4.4 Conclusion

The rapid growth in the global use of ART warrants continued evaluation of its impact on mental health symptom trajectories. This systematic review demonstrates that anxiety symptoms following ART conception compared to SC are varied, with some studies revealing heightened pregnancy-specific anxieties in ART populations and others indicating similar or lower anxiety symptomatology. Further, findings differed by type of assessment, gestational stage, and psychosocial contexts. Such nuanced and mixed results underscore the need for longitudinal investigations that track perinatal anxiety trajectories from conception through the postpartum period. In addition to providing clinical value, clarifying varying symptom pathways may provide insight into potential intergenerational consequences for offspring well-being.

## Data Availability

The original contributions presented in the study are included in the article/**Supplementary Material**. Further inquiries can be directed to the corresponding author.

## Footnotes

1 Throughout this review, the term “pregnant women" will be used to discuss pregnancy in connection to individuals who identify as female to maintain consistency with the nomenclature most often used in the studied literature. However, not all individuals who conceive using ART identify as female, and additional research should be conducted to better understand the unique experiences and specific challenges of non-female birthing parents.

## References

[1] Pinborg A Wennerholm U-B Bergh C . Long-term outcomes for children conceived by assisted reproductive technology. Fertil Steril. (2023) 120:449–56. doi: 10.1016/j.fertnstert.2023.04.022 37086833

[2] Grainger DA Tjaden BL . Assisted reproductive technologies. In: Women and Health. Amsterdam, Netherlands: Elsevier (2000). p. 215–225b. doi: 10.1016/B978-012288145-9/50020-6

[3] Sunderam S . Assisted reproductive technology surveillance—United State. MMWR. Surveillance. Summaries. (2022) 71. doi: 10.15585/mmwr.ss7104a1 35176012 PMC8865855

[4] Mertens J Belva F Van Montfoort APA Regin M Zambelli F Seneca S . Children born after assisted reproduction more commonly carry a mitochondrial genotype associating with low birthweight. Nat Commun. (2024) 15:1232. doi: 10.1038/s41467-024-45446-1 38336715 PMC10858059

[5] Lazzari E Potančoková M Sobotka T Gray E Chambers GM . Projecting the contribution of assisted reproductive technology to completed cohort fertility. Popul. Res Policy Rev. (2023) 42:6. doi: 10.1007/s11113-023-09765-3 36789330 PMC9912242

[6] Raja NS Russell CB Moravek MB . Assisted reproductive technology: Considerations for the nonheterosexual population and single parents. Fert. Steril. (2022) 118:47–53. doi: 10.1016/j.fertnstert.2022.04.012 35610093

[7] Rebar RW . What are the risks of the assisted reproductive technologies (ART) and how can they be minimized? Reprod Med Biol. (2013) 12:151–8. doi: 10.1007/s12522-013-0156-y 29699141 PMC5904622

[8] eClinicalMedicine . The current status of IVF: Are we putting the needs of the individual first? eClinicalMedicine. (2023) 65. doi: 10.1016/j.eclinm.2023.102343 38106562 PMC10725012

[9] Herbert DL Lucke JC Dobson AJ . Depression: An emotional obstacle to seeking medical advice for infertility. Fert. Steril. (2010) 94:1817–21. doi: 10.1016/j.fertnstert.2009.10.062 20047740

[10] Yakupova VA Zakharova EI Abubakirov AN . The mental state of women with an IVF pregnancy. Psychol Russia.: State. Art. (2015) 8:14. doi: 10.11621/pir.2015.0102

[11] Chen T-H Chang S-P Tsai C-F Juang K-D . Prevalence of depressive and anxiety disorders in an assisted reproductive technique clinic. Hum Reprod. (2004) 19:2313–8. doi: 10.1093/humrep/deh414 15242992

[12] Liu Y-F Fu Z Chen S-W He X-P Fan L-Y . The analysis of anxiety and depression in different stages of *in vitro* fertilization-embryo transfer in couples in China. Neuropsychiatr Dis Treat. (2021) Volume 17:649–57. doi: 10.2147/NDT.S287198 33658786 PMC7920591

[13] An Y Sun Z Li L Zhang Y Ji H . Relationship between psychological stress and reproductive outcome in women undergoing *in vitro* fertilization treatment: Psychological and neurohormonal assessment. J Ass. Reprod Genet. (2013) 30:35–41. doi: 10.1007/s10815-012-9904-x 23212833 PMC3553357

[14] Anderheim L Holter H Bergh C Möller A . Does psychological stress affect the outcome of *in vitro* fertilization? Hum Reprod. (2005) 20:2969–75. doi: 10.1093/humrep/dei219 16123098

[15] Bagade T Thapaliya K Breuer E Kamath R Li Z Sullivan E . Investigating the association between infertility and psychological distress using Australian Longitudinal Study on Women’s Health (ALSWH). Sci Rep. (2022) 12:10808. doi: 10.1038/s41598-022-15064-2 35752691 PMC9233676

[16] Huang M-Z Kao C-H Lin K-C Hwang J-L Puthussery S Gau M-L . Psychological health of women who have conceived using assisted reproductive technology in Taiwan: Findings from a longitudinal study. BMC Women’s. Health. (2019) 19:97. doi: 10.1186/s12905-019-0801-7 31299964 PMC6626344

[17] Faa G Manchia M Fanos V . Assisted reproductive technologies: A new player in the foetal programming of childhood and adult diseases? Pediatr Rep. (2024) 16:329–38. doi: 10.3390/pediatric16020029 38804372 PMC11130896

[18] Luke B Brown MB Wantman E Forestieri NE Browne ML Fisher SC . The risk of birth defects with conception by ART. Hum Reprod. (2021) 36:116–29. doi: 10.1093/humrep/deaa272 33251542 PMC8679367

[19] Kadir RA Veleva Z . Assisted reproductive technology and multiple pregnancy. In: D’Angelo A Amso NN , editors. Ultrasound in Assisted Reproduction and Early Pregnancy, 1st. Boca Raton, FL: CRC Press (2020). p. 190–7. doi: 10.1201/9781351046237-15

[20] Grigoriadis S Graves L Peer M Mamisashvili L Tomlinson G Vigod SN . Maternal anxiety during pregnancy and the association with adverse perinatal outcomes: Systematic review and meta-analysis. J Clin Psychiatry. (2018) 79:17r12011. doi: 10.4088/JCP.17r12011 30192449

[21] Barker D . The fetal origins of adult disease. Fetal. Maternal Med Rev. (1994) 6:71–80. doi: 10.1017/S0965539500001005 41292463

[22] Beydoun H Saftlas AF . Physical and mental health outcomes of prenatal maternal stress in human and animal studies: A review of recent evidence. Paed. Perinatal. Epidemiol. (2008) 22:438–66. doi: 10.1111/j.1365-3016.2008.00951.x 18782252

[23] Lipner E Murphy SK Ellman LM . Prenatal maternal stress and the cascade of risk to schizophrenia spectrum disorders in offspring. Curr Psychiatry Rep. (2019) 21:99. doi: 10.1007/s11920-019-1085-1 31522269 PMC7043262

[24] Pike MR Lipner E O'Brien KJ Breen EC Cohn BA Cirillo PM . Prenatal maternal inflammation, childhood cognition and adolescent depressive symptoms. Brain Behav Immun. (2024) 119:908–18. doi: 10.1016/j.bbi.2024.05.012 PMC11844254 38761818

[25] Wadhwa PD Sandman CA Garite TJ . The neurobiology of stress in human pregnancy: implications for prematurity and development of the fetal central nervous system. Prog Brain Res. (2001) 133:131–42. doi: 10.1016/S0079-6123(01)33010-8 11589126

[26] Barker ED Jaffee SR Uher R Maughan B . The contribution of prenatal and postnatal maternal anxiety and depression to child maladjustment. Depression Anxiety. (2011) 28:696–702. doi: 10.1002/da.20856 21769997

[27] Polte C Junge C Von Soest T Seidler A Eberhard-Gran M Garthus-Niegel S . Impact of maternal perinatal anxiety on social-emotional development of 2-year-olds, a prospective study of Norwegian mothers and their offspring: The impact of perinatal anxiety on child development. Maternal Child Health J. (2019) 23:386–96. doi: 10.1007/s10995-018-2684-x 30610530

[28] Capron LE Glover V Pearson RM Evans J O’Connor TG Stein A . Associations of maternal and paternal antenatal mood with offspring anxiety disorder at age 18 years. J Affect Disord. (2015) 187:20–6. doi: 10.1016/j.jad.2015.08.012 26301478 PMC4595479

[29] Si S Zhao G Song G Liu J . Assisted reproductive technologies and postpartum depressive symptoms: A meta-analysis. J Affect Disord. (2024) 356:300–6. doi: 10.1016/j.jad.2024.03.168 38583599

[30] Matthiesen SMS Frederiksen Y Ingerslev HJ Zachariae R . Stress, distress and outcome of assisted reproductive technology (ART): A meta-analysis. Hum Reprod. (2011) 26:2763–76. doi: 10.1093/humrep/der246 21807816

[31] Purewal S Chapman SCE van den Akker OBA . Depression and state anxiety scores during assisted reproductive treatment are associated with outcome: A meta-analysis. Reprod BioMed. Online. (2018) 36:646–57. doi: 10.1016/j.rbmo.2018.03.010 29622404

[32] Milazzo A Mnatzaganian G Elshaug AG Hemphill SA Hiller JE Group, on behalf of T. A. H. S . Depression and anxiety outcomes associated with failed assisted reproductive technologies: A systematic review and meta-analysis. PloS One. (2016) 11:e0165805. doi: 10.1371/journal.pone.0165805 27835654 PMC5106043

[33] Gourounti K . Psychological stress and adjustment in pregnancy following assisted reproductive technology and spontaneous conception: A systematic review. Women Health. (2016) 56:98–118. doi: 10.1080/03630242.2015.1074642 26212077

[34] Veritas Health Innovation . Covidence Systematic Review Software. Melbourne: Veritas Health Innovation (2024). Available online at: https://www.covidence.org (Accessed June 2024). Computer software.

[35] Effective Public Health Practice Project (EPHPP) . Mcmaster Evidence Review & Synthesis Team. Melbourne, Australia. Available online at: https://merst.healthsci.mcmaster.ca/ephpp/ (Accessed June 2024).

[36] Sterne J . (2010). “ An overview of meta-analysis in Stata”, in: United Kingdom Stata Users’ Group Meetings 2010 ( Stata Users Group). Available online at: https://ideas.repec.org/p/boc/usug10/11.html (Accessed June 2024).

[37] Fisher J Wynter K Hammarberg K McBain J Gibson F Boivin J . Age, mode of conception, health service use and pregnancy health: A prospective cohort study of Australian women. BMC Pregnancy. Childbirth. (2013) 13:88. doi: 10.1186/1471-2393-13-88 23565589 PMC3622566

[38] García-Blanco A Diago V Hervás D Ghosn F Vento M Cháfer-Pericás C . Anxiety and depressive symptoms, and stress biomarkers in pregnant women after *in vitro* fertilization: A prospective cohort study. Hum Reprod. (2018) 33:1237–46. doi: 10.1093/humrep/dey109 29796614

[39] Harf-Kashdaei E Kaitz M . Antenatal moods regarding self, baby, and spouse among women who conceived by *in vitro* fertilization. Fert. Steril. (2007) 87:1306–13. doi: 10.1016/j.fertnstert.2006.11.035 17368452

[40] Hjelmstedt A Widström A-M Wramsby H Collins A . Patterns of emotional responses to pregnancy, experience of pregnancy and attitudes to parenthood among IVF couples: A longitudinal study. J Psy. Obstet. Gynecol. (2003) 24:153–62. doi: 10.3109/01674820309039669 14584302

[41] Hjelmstedt A Widström A-M Collins A . Psychological correlates of prenatal attachment in women who conceived after *in vitro* fertilization and women who conceived naturally. Birth. (2006) 33:303–10. doi: 10.1111/j.1523-536X.2006.00123.x 17150069

[42] Klock SC Greenfeld DA . Psychological status of *in vitro* fertilization patients during pregnancy: A longitudinal study. Fert. Steril. (2000) 73:1159–64. doi: 10.1016/S0015-0282(00)00530-6 10856475

[43] McMahon CA Boivin J Gibson FL Hammarberg K Wynter K Saunders D . Pregnancy-specific anxiety, ART conception and infant temperament at 4 months post-partum. Hum Reprod. (2013) 28:997–1005. doi: 10.1093/humrep/det029 23427229

[44] McMahon CA Ungerer JA Beaurepaire J Tennant C Saunders D . Anxiety during pregnancy and fetal attachment after in-vitro fertilization conception. Hum Reprod. (1997) 12:176–82. doi: 10.1093/humrep/12.1.176 9043925

[45] Pellerone M Martinez-Torvisco J Razza SG Commodari E Miccichè S . Precursors of prenatal attachment and anxiety during pregnancy in women who procreate naturally and pregnant women following assisted reproduction technology. Int J Environ Res Public Health. (2023) 20:6945. doi: 10.3390/ijerph20206945 37887682 PMC10606198

[46] Poikkeus P Saisto T Unkila-Kallio L Punamaki RL Repokari L Vilska S . Fear of childbirth and pregnancy-related anxiety in women conceiving with assisted reproduction. Obstet. Gynecol. (2006) 108:70–6. doi: 10.1097/01.AOG.0000222902.37120.2f 16816058

[47] Raguz N McDonald SW Metcalfe A O’Quinn C Tough SC . Mental health outcomes of mothers who conceived using fertility treatment. Reprod Health. (2014) 11:19. doi: 10.1186/1742-4755-11-19 24581007 PMC3996036

[48] Ranjbar F Warmelink C Mousavi R Gharacheh M . Maternal-fetal attachment and anxiety in pregnant women who conceived through assisted reproductive technology: A longitudinal study. Int J Reprod BioMed. (IJRM.). (2021), 1075–84. doi: 10.18502/ijrm.v19i12.10058 35098009 PMC8792377

[49] Sälevaara M Punamäki R Unkila‐Kallio L Vänskä M Tulppala M Tiitinen A . The mental health of mothers and fathers during pregnancy and early parenthood after successful oocyte donation treatment: A nested case‐control study. Acta Obstet. Gynecol. Scandinavica. (2018) 97:1478–85. doi: 10.1111/aogs.13421 29975790

[50] Salih Joelsson L Tydén T Wanggren K Georgakis MK Stern J Berglund A . Anxiety and depression symptoms among sub-fertile women, women pregnant after infertility treatment, and naturally pregnant women. Eur Psychiatry. (2017) 45:212–9. doi: 10.1016/j.eurpsy.2017.07.004 28957789

[51] Saravanan V Desai G Satyanarayana VA . Antenatal predictors of postnatal maternal attachment and competence after assisted conception—a prospective cohort study in South India. Arch Women’s. Ment Health. (2023) 26:549–60. doi: 10.1007/s00737-023-01340-1 37393349

[52] Simoni MK Gilstad-Hayden K Naqvi SH Pal L Yonkers KA . Progression of depression and anxiety symptoms in pregnancies conceived by assisted reproductive technology in the United States. J Psy. Obstet. Gynecol. (2022) 43:214–23. doi: 10.1080/0167482X.2021.1971193 34472405 PMC10116357

[53] Szemes Z Talabér J Bachorecz M Baji I . Effects of assisted reproductive treatments on pregnant women’s mental health. New Med. (2014) 2014:151–5.

[54] Tendais I Figueiredo B . Parents’ anxiety and depression symptoms after successful infertility treatment and spontaneous conception: Does singleton/twin pregnancy matter? Hum Reprod. (2016) 31:2303–12. doi: 10.1093/humrep/dew212 27609986

[55] Vilska S Unkila-Kallio L Punamaki R-L Poikkeus P Repokari L Sinkkonen J . Mental health of mothers and fathers of twins conceived via assisted reproduction treatment: A 1-year prospective study. Hum Reprod. (2009) 24:367–77. doi: 10.1093/humrep/den427 19043082

[56] Spielberger CD Gorsuch RL Lushene RE Vagg PR Jacobs GA . Manual for the State-Trait Anxiety Inventory (Form Y). Palo Alto, CA: Consulting Psychologists Press (1983).

[57] Gashi A . The impact of assisted reproductive technology (Art), to the increasing incidence of the high order multiple pregnancies. Gynecol. Obstet. (2015) 05. doi: 10.4172/2161-0932.1000302

[58] Zuo L Fan Y Ai J Tian L . Analysis of factors related to early miscarriage after *in vitro* fertilization embryo transfer. Gynecol. Obstet. Clin Med. (2022) 2:171–4. doi: 10.1016/j.gocm.2022.09.001 38826717

[59] Antoniou E Tzanoulinou M-D Stamoulou P Orovou E . The important role of partner support in women’s mental disorders during the perinatal period. A literature review. Maedica. - A J Clin Med. (2022) 17. doi: 10.26574/maedica.2022.17.1.194 35733735 PMC9168558

[60] Khademi K Kaveh MH . Social support as a coping resource for psychosocial conditions in postpartum period: A systematic review and logic framework. BMC Psychol. (2024) 12:301. doi: 10.1186/s40359-024-01814-6 38807228 PMC11131291

[61] Hidalgo-Padilla L Toyama M Zafra-Tanaka JH Vives A Diez-Canseco F . Association between maternity leave policies and postpartum depression: A systematic review. Arch Women’s. Ment Health. (2023) 26:571–80. doi: 10.1007/s00737-023-01350-z 37458837 PMC10491689

[62] Ankerstjerne LBS Laizer SN Andreasen K Normann AK Wu C Linde DS . Landscaping the evidence of intimate partner violence and postpartum depression: A systematic review. BMJ Open. (2022) 12:e051426. doi: 10.1136/bmjopen-2021-051426 35584869 PMC9119188

[63] Casey P Goolsby S Berkowitz C Frank D Cook J Cutts D . Maternal depression, changing public assistance, food security, and child health status. Pediatrics. (2004) 113:298–304. doi: 10.1542/peds.113.2.298 14754941

[64] Pourhoseini SA Akbary A Mahmoudi H Akbari M Heydari ST . Association between prenatal period exposure to ambient air pollutants and development of postpartum depression: A systematic review and meta-analysis. Int J Environ Health Res. (2022) 34:455–65. doi: 10.1080/09603123.2022.2153808 36469809

[65] Sun Y Headon KS Umer W Jiao A Slezak JM Avila CC . Association of postpartum temperature exposure with postpartum depression: A retrospective cohort study in Southern California. Environ Health Perspect. (2024) 132:117004. doi: 10.1289/EHP14783 39601565 PMC11601096

[66] Cesta CE Viktorin A Olsson H Johansson V Sjölander A Bergh C . Depression, anxiety, and antidepressant treatment in women: Association with *in vitro* fertilization outcome. Fert. Steril. (2016) 105:1594–1602.e3. doi: 10.1016/j.fertnstert.2016.01.036 26920258

[67] Sejbaek CS Hageman I Pinborg A Hougaard CO Schmidt L . Incidence of depression and influence of depression on the number of treatment cycles and births in a national cohort of 42,880 women treated with ART. Hum Reprod (Oxford. England.). (2013) 28:1100–9. doi: 10.1093/humrep/des442 23300199

[68] Egsgaard S Bliddal M Jølving LR Liu X Sonne H Munk-Olsen T . The association between medically assisted reproduction and postpartum depression: A register-based cohort study. BJOG.: Int J Obstet. Gynaecol. (2025) 132:991–9. doi: 10.1111/1471-0528.18127 40097347 PMC12051253

[69] Barber GA Steinberg JR . The association between pregnancy intention, fertility treatment use, and postpartum depression. Soc Sci Med. (2022) 314:115439. doi: 10.1016/j.socscimed.2022.115439 36274452

[70] Smith CA De Lacey S Chapman M Ratcliffe J Norman RJ Johnson NP . The effects of acupuncture on the secondary outcomes of anxiety and quality of life for women undergoing IVF: A randomized controlled trial. Acta Obstet. Gynecol. Scandinavica. (2019) 98:460–9. doi: 10.1111/aogs.13528 30592302

[71] Whynott RM Summers KM Mejia RB Segre LS Ryan G Pawlak SA . Creating affiliations, learning, and mindfulness for *in vitro* fertilization patients (CALM IVF): A clinical trial. F&S. Rep. (2023) 4:61–71. doi: 10.1016/j.xfre.2023.01.002 36959953 PMC10028477

[72] Garcia Pombo S Armijio Sánchez A Gómez Manzorro MD Ibañez Alonso JA Mantrana Bermejo E Tirado Carillo MDP . P-580 Virtual reality as tool to reduce anxiety during embryo transfer. Hum Reprod. (2023) 38:dead093.912. doi: 10.1093/humrep/dead093.912

[73] Maehara K Iwata H Kimura K Mori E . Experiences of transition to motherhood among pregnant women following assisted reproductive technology: A qualitative systematic review. JBI. Evidence. Synthesis. (2022) 20:725–60. doi: 10.11124/JBIES-20-00545 34410230

